# Patient safety attitude among healthcare workers at different levels of healthcare in Sharqia Governorate, Egypt

**DOI:** 10.4102/phcfm.v14i1.3307

**Published:** 2022-02-24

**Authors:** Yasmin H.H. Hussein, Seham M. Eldeeb, Raghda A. Elshamy, Rasha M.B. Eldin

**Affiliations:** 1 Department of Family Medicine, Faculty of Medicine, Zagazig University, Zagazig, Egypt; 2 Department of Public Health and Community Medicine, Faculty of Medicine, Zagazig University, Zagazig, Egypt; 3 Department of Occupational Medicine, Faculty of Medicine, Zagazig University, Zagazig, Egypt

**Keywords:** burnout, management, safety climate, safety culture, stress

## Abstract

**Background:**

Patient safety (PS) has been identified as a significant healthcare challenge. A good safety attitude helps healthcare workers (HCWs) to decrease medical errors.

**Aim:**

This study aimed to assess the PS attitude and identify its determinants among HCWs.

**Setting:**

This study was conducted in Sharqia Governorate at different levels of health care.

**Methods:**

This was a comparative cross-sectional study that involved240 HCWs selected after using a multistage cluster sampling technique from Sharqia Governorate.In ordertto assess the respondents’ attitudes towards PS, the modified Chinese Safety Attitudes Questionnaire (CSAQ) was used.

**Results:**

The scale with the highest percentage of positive responses, on average, was *safety climate* (49.59%). The study found a statistically significant association between the level of health care and mean scores of ‘teamwork climate, perception of management, job satisfaction, working conditions, and stress recognition’ and the overall CSAQ score. In regression analysis, the highest degree of education and job type were significant predictors of PS attitude among the HCWs under study (*p* = 0.031 and 0.011, respectively).

**Conclusion:**

According to the study’s findings, PS is low among HCWs in both healthcare units and hospitals, with a significantly higher score among hospital workers than among primary care workers. All PS composites need improvement starting with regular assessment of PS culture along with continuous monitoring.

## Introduction

Patient safety (PS) is a worldwide public health topic. According to the World Health Organization, unsafe medical care leads to disabling injuries or deaths in millions of patients.^1^

Patient safety is the prevention and avoidance of patient injuries or adverse events caused by delivery processes of healthcare workers (HCWs).^2^ The safety culture of the organisation serves as a guide on how employees should perform in the workplace, and their behaviour will be influenced or determined by which behaviours are rewarded and acceptable in the workplace. Organisational positive attitude culture is characterised by trust-based communications, shared perceptions of the importance of safety and faith in the efficacy of preventive measures.^3^

Despite the importance of PS in health care, only few organisations have assessed how well their staff culture promotes PS. Assessing the current safety attitudes enables organisations to gain a clear picture of PS issues that require immediate attention, address the strengths and weaknesses of their safety culture, and improve continuous quality management.^4^

Positive attitudes of PS can be enhanced by targeted training as well as creating an openness of workplace culture, being aware of potential hazards and changing behaviour. This should include an approach to mistakes that is open for learning and development, adapting a non-punitive response to error, which deals with errors as an opportunity to learn.^5^

Patient safety is a major issue by health policymakers in many Arab countries, necessitating the identification and analysis of factors that contribute to its occurrence.^6^ According to the Organisation for Economic Cooperation and Development report, the situation in low- and middle-income countries is direr, with approximately 2.6 million deaths occurring because of 134 million adverse events occurring in hospitals each year.^7^ In Africa, information is limited about the scope of PS culture. The study in Ethiopia showed a very low-positive PS grade.^8^ Patient safety in Kenya remains a challenge, with an increasing number of medical errors being reported in the media.^9^ South Africa is among the developing countries that has a higher percentage of PS incidents.^10^ Moreover, Eastern Mediterranean and African Study found that unsafe care affects around 10% of patients, most of those incidents were preventable.^11^

Patient safety requires knowledge and skills in various areas, including human factors and system management, because most preventable errors, such as medication errors, investigation errors and nosocomial infections, are related to these areas.^12^ Patient safety attitude is a significant key influencing factor for improving physician–patient relationship and the quality of service that patients obtain. Reviewing PS attitude regularly enables the organisation management council to realise and monitor the progress of PS within the healthcare processes. In Egypt, different studies highlighted the need for improving the PS culture among healthcare providers. Moreover, relevant research is needed to evaluate the PS attitudes and awareness. This study was designed to assess PS attitude and identify its determinants among HCWs in Sharqia Governorate at various levels of care, including primary health care (PHC) units (primary level) and Zagazig General Hospital (tertiary level). Also, the findings of this study may also be used as an input for the Ministry of Health and population (MOHP) and administrators of the hospitals and healthcare units to make informed decisions regarding PS issues at the study site and in other similar sites in Egypt.

## Methods

### Study design and setting

A comparative cross-sectional study was carried out from 01 October 2020 to the end of March 2021 in Sharqia Governorate at various levels of care (primary and tertiary levels). Sharqia is the third governorate in population at the level of Egypt, where its estimated population is approximately 8 million. It divided administratively into 19 health districts; Zagazig is the capital of Sharqia Governorate.

### Study population

Healthcare workers include physicians, dentists, pharmacists, nurses and technicians, working in PHC units in Zagazig Health District (primary level) and Zagazig General Hospital (tertiary level) for more than six months.

### Eligibility criteria

#### Inclusion criteria

All HCWs working in the places of the study for more than six months and who were willing to participate in the study were included.

#### Exclusion criteria

Healthcare workers working in the places of the study for less than six months and who were on annual leave and sick leave during data collection were excluded from the study.

### Sample size and sampling

The sample size was calculated using Epi Info™,^13^ assuming that the prevalence of positive attitude among HCWs towards teamwork climate is 21.5% at the primary level^14^ and 38% at the tertiary level of health care.^3^ The total sample was 240 with 95% confidence level, and the study power was 80%. As the ratio was 1:1, 120 HCWs each from the primary and tertiary levels of health care were selected to participate in the study.

Using a multistage cluster sampling technique, Zagazig was selected to represent Sharqia Governorate. The Zagazig Health District includes 46 PHC units, from which 20 units were selected randomly to represent the primary level of healthcare and one general hospital (Zagazig General Hospital) consisting of 147 beds was selected to represent the tertiary level of healthcare.

According to Zagazig health directorate records, the total number of HCWs at the Zagazig PHC units and the Zagazig General Hospital in 2020 is 745 and 588, respectively.

The sample units in each group (*n* = 120) were divided in to three subgroups:

- Subgroup A: 40 physicians.
- Subgroup B: 40 pharmacists and dentists.
- Subgroup C: 40 nurses and technicians.

Sample units in subgroups B and C were divided and selected using the proportional allocation method.

A stratified random sampling technique was used to select the study subjects from the selected units and hospital. During this, primarily, the lists of HCWs were obtained from units and hospital. Then, based on the list obtained, a lottery method was used to select the study participants. Next, the objective of the study was explained to them. Finally, self-administered questionnaires were distributed for those HCWs who were willing to participate in the study.

### Tool and data collection

The modified Chinese Safety Attitudes Questionnaire (CSAQ), developed by the Taiwan Joint Commission on Hospital Accreditation, was used in this study.^15^ This tool consists of seven scales: *teamwork climate*, *safety climate*, *job satisfaction*, *stress recognition*, *perception of management*, *working conditions* and *burnout*. The tool consisted of 40 statements, with responses rated using a 5-point Likert scale, indicating the participants’ level of agreement with the statement (i.e., 1 = strongly disagree, 2 = disagree, 3 = neutral, 4 = agree and 5 = strongly agree). During data analysis, the scores of negatively worded items were reversed so that higher scores in the dataset indicated a more positive assessment of the unit’s PS culture. Some demographic characteristics were also collected, including age, sex, marital status, working place, highest degree of education, job title, work shift, years of work experience and previous training on PS.

#### Content validity and reliability

The questionnaire was translated into Arabic and was translated back to English by language experts. The reliability of the scales was tested through internal consistency measurement. It demonstrated an excellent level of reliability (Cronbach’s alpha = 0.915).^16^ One month before the start of this study, a pilot study was conducted to demonstrate any data collection difficulties, evaluate the questionnaire validity and reliability after translation, and estimate the time needed for data collection and expected frequency. No changes were employed, so the pilot sample was included in the main sample.

### Statistical analysis

The Statistical Package for the Social Sciences, version 25,^17^ was used to analyse the data collected. Tables were used to present data as frequencies, proportions, means and standard deviations. Independent samples *t*-tests and one-way analysis of variance were used, as appropriate, to compare PS attitudes at various levels of health care and assess the relationship between participant characteristics and the total mean score of the PS attitude. To identify predictors, linear regression analyses were performed.

### Ethical considerations

After revising the study protocol, the Zagazig University Institutional Review Board (ZU-IRB) granted approval (ZU-IRB #6765). Before the interview, the study’s nature and purpose were explained to participants, and verbal consent was obtained. The information provided by all participants was kept private. The study was approved by the Zagazig health directorate. An official permission letter was obtained from the authority and directed to Zagazig General Hospital and the PHC units included in the study.

## Results

This study included 240 participants, including 120 primary HCWs (PHCWs) (i.e., 40 physicians, 32 nurses, 28 pharmacists, 12 dentists and 8 technicians) and 120 tertiary HCWs (THCWs) (i.e., 40 physicians, 24 nurses, 32 pharmacists, 8 dentists and 16 technicians). The mean age of the PHCWs was 34.7 ± 6.5 years, and that of the THCWs was 35.7 ± 7.8 years. Most respondents were females, were married, did not attend any PS training courses and had direct contact with patients. More than half of them had morning work shifts and worked in the current place for ≥ 6 years. Almost one-third of the PHCWs had university education and working experience ranging from 5 years to < 10 years. The THCWs had high school education (31.7%) and university education (31.7%) and had working experience ranging from 10 years to < 15 years (Table 1).

**TABLE 1:** Basic characteristics of the studied healthcare workers.

Item	Primary care (*n* = 120)		Tertiary care (*n* = 120)	
	*n*	%	*n*	%
**Age (years)**				
Mean ± s.d.	34.7 ± 6.5	-	35.7 ± 7.8	-
**Gender**				
Male	16	13.3	24	20.0
Female	104	86.7	96	80.0
**Marital status**				
Single†	24	20.0	30	25.0
Married	96	80.0	90	75.0
**The highest degree of education**				
High school	26	21.7	38	31.7
University	52	43.3	38	31.7
Master’s degree	32	26.7	24	20
Fellowship	4	3.3	4	3.3
MD degree	6	5.0	16	13.3
**Job type**				
Physician	40	33.3	40	33.3
Nurse	32	26.7	24	20.0
Pharmacist	28	23.3	32	26.7
Dentist	12	10.0	8	6.7
Technician	8	6.7	16	13.3
**Work-shift**				
Morning	76	63.3	64	53.3
Mixed	44	36.7	56	46.7
**Working experience (years)**				
< 5	12	10.0	16	13.4
5 to <10	48	40.0	40	33.3
10 to < 15	32	26.7	40	33.3
≥ 15	28	23.3	24	20.0
**Time working in the current place**				
≤ 1 year	6	5.0	4	3.3
2–5 years	32	26.7	40	33.3
≥ 6 years	82	68.3	76	63.3
**Had any training about patient safety**				
Yes	30	25.0	22	18.3
No	90	75.0	98	81.7
**Form of contact with the patient**				
Direct	92	76.6	90	75.0
Indirect	28	23.3	30	25.0

s.d., standard deviation; MD, doctor of medicine.

† , Single, divorced, widow.

**FIGURE 1:**
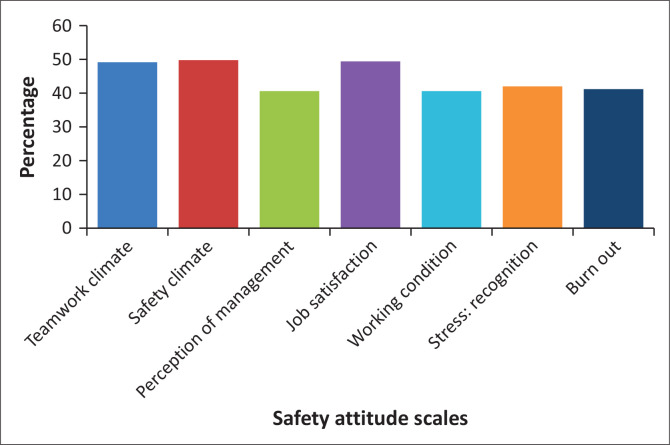
Average of percentage positive responses per Chinese safety attitudes questionnaire scales.

Table 2 displays the participants’ mean scores for each item on the seven PS scales, as well as the percentage of positive responses. The scale with the most positive responses was *safety climate* 49.59%, which was followed by *job satisfaction* 49.5%, t*eamwork climate* 49.17%, *stress recognition* (41.8%), *burnout* (41%), *perception of management* (40.63%) and *working conditions* (40.6%). The average percentage of positive responses per the CSAQ is shown in Figure 1.

**TABLE 2:** Patient safety scales’ mean score and the percentages of positive responses of all participants.

Scale	Mean	s.d.	% of positive responses
**Teamwork climate**			
In this health unit/hospital, it is difficult to speak up if I perceive a problem with patient care.	3.28	1.22	43.3
The physicians and nurses here work together as a well-coordinated team.	2.68	1.21	25.0
Disagreements in this health unit/hospital are appropriately resolved.	3.32	1.28	55.0
Nurse input is well received in this clinical area.	3.35	1.13	56.7
I have the support I need from other personnel to care for patients.	3.65	0.99	69.2
It is easy for personnel in this office to ask questions when there is something that they do not understand.	3.23	1.18	45.8
**Safety climate**			
I am encouraged by my colleagues to report any patient safety concerns I may have.	3.33	1.01	52.0
The culture in this office makes it easy to learn from errors of others.	3.48	1.09	68.0
Medical errors are handled appropriately in this health unit/hospital.	3.27	0.96	43.0
I know the proper channels to direct questions regarding patient safety in this office.	3.28	1.04	48.0
I receive appropriate feedback about my performance.	3.3	0.99	47.0
I would feel safe being treated here as a patient.	3.1	1.17	48.3
In this office, it is difficult to discuss errors.	3.15	1.15	40.8
**Perception of management**			
Senior management of this office is doing a good job.	3.3	1.06	51.7
The management of this office supports my daily efforts.	3.12	1.04	36.7
I am provided with adequate, timely information about events in the hospital that might affect my work.	2.77	1.20	31.6
The levels of staffing in this office are sufficient to handle the number of patients.	3.04	1.14	42.5
**Job satisfaction**			
This health unit/hospital is a good place to work.	3.17	1.29	49.1
I am proud to work in this health unit/hospital.	3.29	1.03	47.5
Working in this place is like being part of a large family.	3.36	0.92	44.2
Morale in this clinical area is high.	3.39	1.08	53.3
I like my job.	3.33	1.15	53.4
**Working condition**			
This health unit/hospital does a good job of training new personnel.	3.85	1.05	75.0
This health unit/hospital constructively deals with problem physicians and employees.	3.05	1.18	45.0
All the necessary information for diagnostic and therapeutic decisions is routinely available to me.	2.66	1.06	21.6
Trainees in my discipline are adequately supervised.	3.02	0.99	20.8
**Stress recognition**			
When my workload becomes excessive, my performance is impaired.	3.09	1.16	41.7
I am more likely to make errors in tense or hostile situations.	3.17	1.15	43.3
Fatigue impairs my performance during emergency situations.	3.10	1.14	33.2
I am less effective at work when I am fatigued.	3.24	1.16	49.0
**Burn out**			
I feel like I’m at the end of my rope.	2.88	1.18	35.8
I feel burned out from my work.	3.19	1.12	43.3
I feel frustrated by my job.	3.18	1.19	45.8
I feel I’m working too hard on my job.	3.29	1.38	50.8
I feel emotionally drained from my work.	3.33	1.18	54.2
I feel used up at the end of the work day.	2.71	1.12	26.7
I feel fatigued when I get up in the morning and have to face another day on the job.	2.89	1.13	31.5
Working with people all day is really a strain for me.	3.18	1.16	45.9
Working with people directly puts too much stress on me.	2.94	1.13	35.0

s.d., standard deviation.

There were significant statistical association between the level of health care and the mean scores of *Teamwork climate, Perception of management, Job satisfaction, working condition, Stress recognition* and the overall CSAQ score (*p* = < 0.001, 0.003, < 0.001, 0.002, < 0.001, 0.001, respectively), as the THCWs had a significantly higher mean score (Table 3). The total mean score of PS attitude was significantly higher among those aged ≥ 40 years (132.98 ± 25.12; *p* < 0.001), male respondents (131.90 ± 30.65; *p* = 0.009), married (125.79 ± 20.64; *p* = 0.014), MD educated (143.50 ± 14.92, *p* = < 0.001), nurses (131.00 ± 16.32; *p* = < 0.001) and those who had PS training (130.52 ± 17.75; *p* = 0.005) (Table 4).

**TABLE 3:** Association between the level of health care and the mean scores of patient safety.

Scale	Level of healthcare		*p*	95% lower limit	95% upper limit
	Primary	Tertiary			
Teamwork climate					
Mean ± s.d.	18.68 ± 3.29	20.34 ± 3.86	< 0.001*	2.58	0.75
Safety climate					
Mean ± s.d.	22.98 ± 4.23	22.81 ± 6.47	0.758	0.89	1.23
Perception of management					
Mean ± s.d.	11.68 ± 2.19	12.79 ± 3.41	0.003*	1.84	0.38
Job satisfaction					
Mean ± s.d.	15.41 ± 3.02	17.67 ± 5.09	< 0.001*	3.31	1.19
Working condition					
Mean ± s.d.	11.92 ± 2.81	13.23 ± 3.65	0.002*	2.14	0.49
Stress recognition					
Mean ± s.d.	11.30 ± 2.61	13.90 ± 3.02	< 0.001*	3.32	1.88
Burnout					
Mean ± s.d.	27.45 ± 7.40	27.75 ± 7.89	0.762	2.25	1.65
Overall CSAQ score					
Mean ± s.d.	119.43 ± 17.74	128.50 ± 23.26	0.001*	14.35	3.81

CSAQ, Chinese Safety Attitudes Questionnaire; s.d., standard deviation.

* , Statistically significant.

**TABLE 4:** Association between participants’ characteristics and the total mean score of the patient safety attitude.

Item	Mean ± s.d.	*p*
**Age**		
20–29	121.64 ± 8.05	< 0.001*
30–39	121.99 ± 22.08	
≥ 40	132.98 ± 25.12	
**Gender**		
Male	131.90 ± 30.65	0.009*
Female	122.35 ± 18.33	
**Marital status**		
Single	117.78 ± 21.78	0.014*
Married	125.79 ± 20.64	
**The highest degree of education**		
High school	125.34 ± 18.59	< 0.001*
University	122.01 ± 13.88	
Master’s degree	118.96 ± 30.69	
Fellowship	115.00 ± 11.14	
MD degree	143.50 ± 14.92	
**Job type**		
Physician	124.71 ± 22.41	< 0.001*
Nurse	131.00 ± 16.32	
Pharmacist	121.40 ± 13.19	
Dentist	120.20 ± 41.32	
Technician	114.46 ± 13.24	
**Had any training about patient safety**		
Yes	130.52 ± 17.75	0.005*
No	122.12 ± 21.66	

s.d., standard deviation; MD, doctor of medicine.

* , Statistically significant.

In the regression analysis, educational degree and job type were significant predictors of PS attitude among the HCWs under study. Non-significant R^2^ indicates a good fit model of PS attitude (Table 5).

**TABLE 5:** Linear regression analysis for predictors of positive patient safety attitude.

Independent factors	Coefficient	Standard error	*t*	*p*
Age (≥ 40)	0.951	2.600	0.366	0.715
Gender (Male)	–3.760	4.099	–0.917	0.360
Marital status (Married)	6.171	3.549	1.739	0.083
The highest degree of education (MD)	5.026	2.312	2.174	0.031*
Job type (Nurse)	–4.617	1.805	–2.558	0.011*
Had any training about patient safety (Yes)	5.693	3.972	1.433	0.153

Note: *R* = 0.293, *R*^2^ = 0.086.

MD, doctor of medicine.

* , Statistically significant.

## Discussion

Patient safety is an important aspect of healthcare quality. As healthcare organisations strive to improve, the importance of fostering a safety culture within them is becoming more widely recognised.^18^ This study showed a difference between PHCWs and THCWs regarding the level of education and duration of working experience, which attributed to different structural job distribution between PHC units and hospitals. In terms of previous PS training, approximately 20% have prior training .This result indicates the need for continuously establishing more training programmes on PS issues for all HCWs at all healthcare organisations. This result conforms to that of a study by the Faculty of Medicine of Cairo University.^2^

The overall percentage of positive responses in the current study indicated a poor scale (score ≤ 75%) regarding PS attitude across six domains (40% – 49.6%) and associated with relatively increase of burn-out score (41.4%) because positive safety attitude associated with burn out absence and high ability to handle stressful situations.^19^ This result revealed a negative safety perception among physicians, nurses and laboratory technicians and showed the demanding improvement of all PS domains. Furthermore, this finding reveals the state of PS in the Egyptian health system, as well as most of the developing countries where PS is not given enough attention.

This study’s overall PS score is lower than that stated by the Agency for Healthcare Research and Quality (2014) (64%), Palestine (63.5%), Saudi Arabia (60%), Lebanese private hospitals, (72.5%), and Hospital Survey on Patient Safety Culture (2012) in China (63%) and study in a Kenya (65.8%).^9,20,21,22^ One of the possible explanations for the disparity between the results of this study and those of other studies is a lack of PS culture awareness, training programmes for HCWs and institutional performance improvement strategies.

In this study, the *safety climate* score was higher than those of other domains, implying that creating a non-punitive culture, developing prompt networks, providing training and motivating HCWs to discuss and report adverse events and timely. However, this scale scored low, indicating the need for more attention to infrastructure and leadership attitudes towards dealing with errors and learning from adverse events.

The *job satisfaction* score was the second highest; the higher response rate was related to the statement, ‘I like my job’, suggesting that when the workplace facilitates HCWs to feel family warmth, several HCWs will love their work and behave well, which would be beneficial to PS.

The *perception of management* and *working conditions* domains were rated lower than other domains, implying that supervising and training new workers and the preservation of diagnostic and therapeutic data would be valuable to PS. Furthermore, to foster a better working environment, we must train and supervise new employees, and ensure that therapeutic information is readily available.^23^

In this study, a significant difference in the mean score of PS attitude was observed between PHCWs and THCWs in terms of *teamwork climate*, *perception of management*, *job satisfaction*, *working conditions* and *stress recognition*. Furthermore, the overall score in the seven PS scales of the CSAQ was higher at the tertiary level than that at the primary level. This finding conforms to that of a study conducted in Turkey, which found that the average overall score of PS culture in PHC units is lower than that of hospitals.^24^ This finding explained why healthcare units do not handle severe clinical cases that can only be handled by intensive care units, emergency departments or hospitals. Because most dangerous medical interventions are performed in hospitals, hospital workers may receive additional training and specialisation in safety-related issues.^25^ This result is expected because PS topics have largely been identified at the hospital level, with less emphasis placed to the primary care level, where adverse outcomes are less common, and the greatest volume of care is delivered with infrastructure restrictions and guidelines and criteria for safe practices.^26^

This study showed that older age groups had higher attitude scores; this may be explained by higher working experiences, which lead to an increase in tolerance to changes in working conditions. Furthermore, it is expected that the increasing age of HCWs will make them more appreciative of their jobs and push them to improve their performance.^27^ Moreover, new workers may be less sensitive to safety issues, according to El Shafei and Zayed’s and Abdi et al.’s studies.^14,24,28^ This study showed that male participants had higher attitude scores than females. This can be explained by difference in specialties and responsibility distribution and duration of work between them.

In this study, a link was observed between previous PS training courses and caregivers’ perceived influence on PS. This result is suspected and related to the fact that education about PS impacts the attitude of healthcare worker which determines how they act and behave. This finding conforms to that stated by Asem et al.,^2^ Zhao et al.,^29^ and Biresaw et al.^30^ in Ethiopia, which reported that nurses who received information on PS were 4.39 times more likely to have good knowledge and attitude as compared with those who had not.

Regarding job type, this study showed that staff holding master’s degree had higher attitude scores than those with lower educational levels, and regression analysis revealed that educational level is a significant predictor, which explained by highly educated health professionals having a broader range of ideas and thinking more about the problems, which may lead to more pressure, negatively affecting teamwork climate.^29^

Another predictor revealed by regression analysis was that nurses had a more positive attitude towards PS than physicians. This finding is as a result of the nurses being the first point of contact with clients for most basic benefit packages and services.^31^ Furthermore, because most nurses were younger and received more information about safety culture, they may be more resilient and could handle stressful working conditions.^32^ The findings conform to those of the studies by Abu-El-Noo and El Shafei and Zayed, who reported relatively high mean scores among nurses, particularly in the *stress recognition* domain, working in PHC centres. In contrast, various studies have revealed that physicians outperformed nurses.^28,33,34^

### Strengths and limitations of the study

The strength of our study was based on the comparison of PS between different levels of care in contrast to other studies. The limitation was that the data collected depended on self-reports from the participants. The accuracy of the questionnaire may have been affected by the fear of punishments. Moreover, we wanted to assess PS culture from the perceptions of other healthcare givers, for example, nursing students, clerks, managers and patients. Furthermore, the cross-sectional nature of the study design does not confirm definitive cause and effect relationship.

## Conclusion

The current study showed that the PS score is low among HCWs at both healthcare units and hospitals, and no domain had a score higher than 75. Moreover, hospital HCWs had significantly higher scores in most PS domains and overall score than PHCWs. Personnel with master’s degree and nurses were predictors of good PS attitude.

All PS domains necessitate improvement beginning with continuous PS culture assessment along with regular monitoring and increasing the awareness of PS culture. Other studies are required in different regions and healthcare settings to generalise the results to other units and hospitals in Egypt.

Training and learning by providing skills for healthcare professionals is a key for optimising quality and PS. Thus, hospitals and healthcare units’ management should be prepared to all new staff and they should be given a general orientation programme and training to outline the policies, procedures, and their role and responsibilities in order to improve quality of patient care. Also, it is necessary to create an open and non-punitive culture to encourage and train health professionals to report adverse events. Moreover, it is necessary to establish scientific and reasonable hierarchical diagnosis and treatment system, arrange health professionals’ workload reasonably, increase the number of health professionals reasonably and reduce the stress of health professionals.

Further research is recommended to include staffing and the work environment factors to predict the outcomes of care.
